# Pediatric Dosing and Body Size in Biotherapeutics

**DOI:** 10.3390/pharmaceutics2040389

**Published:** 2010-12-16

**Authors:** Rong Shi, Hartmut Derendorf

**Affiliations:** Department of Pharmaceutics, University of Florida, Gainesville, FL, 32610, USA; E-Mail: rongshi@ufl.edu

**Keywords:** pediatric, dosing, proteins, peptides

## Abstract

Although pediatric doses for biotherapeutics are often based on patients' body weight (mg/kg) or body surface area (mg/m^2^), linear body size dose adjustment is highly empirical. Growth and maturity are also important factors that affect the absorption, distribution, metabolism and excretion (ADME) of biologics in pediatrics. The complexity of the factors involved in pediatric pharmacokinetics lends to the reconsideration of body size based dose adjustment. A proper dosing adjustment for pediatrics should also provide less intersubject variability in the pharmacokinetics and/or pharmacodynamics of the product compared with no dose adjustment. Biological proteins and peptides generally share the same pharmacokinetic principle with small molecules, but the underlying mechanism can be very different. Here, pediatric and adult pharmacokinetic parameters are compared and summarized for selected biotherapeutics. The effect of body size on the pediatric pharmacokinetics for these biological products is discussed in the current review.

## 1. Introduction

**Figure 1:**
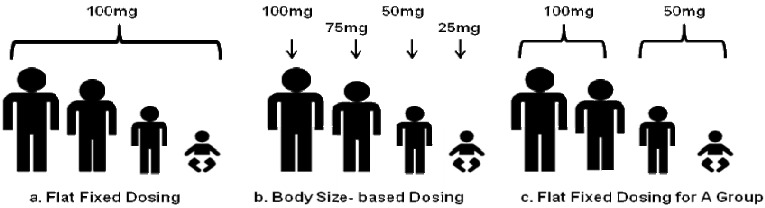
Drug dosing strategies. (**a**) An example of fixed dosing. (**b**) An example of body size-based dosing. (**c**) An example of fixed dosing by different age groups or different body size groups.

Due to the complexity and costs of pediatric safety and efficacy studies, pharmaceutical companies are somewhat reluctant to study drugs and biological products in children. Without safety and efficacy studies in children, physicians are often forced to make empirical assumptions to treat children on a trial-and-error basis [1]. The clinical outcomes of such treatments in children can be promising, marginal or harmful. Physiological development during childhood can produce significant effects on drug absorption, distribution, metabolism and excretion. After birth, the changes in gastrointestinal absorption, secretion, motility, metabolism and transport, as well as first-pass effects will affect the absorption of the drug; the changes in body composition, tissue perfusion and plasma protein binding will affect the distribution of the drug; maturation in cytochrome P450 enzyme-mediated metabolism and phase II metabolism will affect hepatic clearance; and maturation of glomerular filtration and renal tubular function will affect renal clearance [2]. In general, all the effects of maturation on the pharmacokinetics of a given drug are not well understood. Usually, drugs are given with two types of dosing strategies: flat fixed dosing and body size-based dosing (Figure 1 (a) and (b)). The most common dosing approach for pediatrics is body surface area (BSA)/body weight adjusted dosing. Small children are rarely given the same dose as adults (Figure 1a). However, a convenient dosing approach that sometimes provides accurate dosing and less intersubject variability is often overlooked by body size-based dosing. This dosing approach provides a fixed dose for a certain age or certain body size group (Figure 1c). Fixed dosing for a patient group provides quite a few advantages compared to body size-based dosing: ease of preparation and administration, less risk of medical errors, better patient compliance, and cost effectiveness. When body weight or BSA adjusted pharmacokinetic parameters can explain the difference between pediatrics and adults, body weight or BSA dose adjustment can provide comparable exposure in pediatrics as in adults. However, this is not always the situation. More often, the trend and extent of the pharmacokinetic difference between pediatrics and adults across different age groups are not predictable. Clearance and volume of distribution of drugs can be higher, but can also potentially be lower in younger children, compared with older children or adults [3]. Therefore, simply adjusting the pediatric dose according to the body weight/BSA may not be an accurate dosing approach. Age should also be taken into account for the maturation in pediatrics. Sometimes, even taking age into consideration for dose determination, might still not accurately account for all variables related to the different stages of maturation as well as the physiological differences between pediatrics and adults. More importantly, any dose adjustment should decrease the variability in the resulting exposure, which would be proof that it makes sense to apply this dose adjustment. 

### 1.1. General Pharmacokinetics in Pediatrics

The definitions of pharmacokinetics in children are as follows:

Premature: gestational age < 36 weeks
Full-term: gestational age ≥ 36
Neonates: 0–1 months
Infants: 1–12 months
Children: 1–12 years
Adolescent: 12–16 years

Unlike adults, the pharmacokinetics in pediatrics is remarkably affected by the growth and development of children. Body composition and organ function change over childhood development. The total body water, extracellular fluid and intracellular fluid constitution of the body weight in fetuses, premature or full-term neonates, infants, adolescent and adults are shown in Table 1 [4,5]. The total body water and extracellular fluid content decreases dramatically among premature or full-term neonates and infants. Additionally, fat contributes to 3% of the total body weight in premature neonates, and 12% of the total body weight in full-term neonates; and it is more than 20% by the age of 4–5 months. Protein mass in infants before they start walking is around 20% and increases to 40% in adults. Lean muscle tissue contains about 75% water by weight. Therefore, total body water, fat and muscle change at different ages may produce significant changes in volume of distribution and systemic concentration of the drug. Different organs such as the heart, liver, and kidney account for more body weight in children than in adults in percentage (Table 2. [4]). This can explain the cases when infants or children have a faster body weight normalized clearance than adults, since infants or children have a relatively larger liver or kidney per body size compared with adults. The glomerular filtration rate (GFR) is an important factor in drug clearance. Table 3 lists the changes occurring in the GFR and renal plasma flow (RPF) with age. Infants to children from an age of 1–10 days, to one month and six months roughly double the GFR in the three stages [4]. GRF reaches maturation at the age of one year and stays almost constant from the age of one to 70 years. The GRF/vECF (extracellular fluid volume) ratio and GRF/BSA ratio was studied in 130 patients (age range 1–80 years; 40 patients < 12 years). Neither GFR-based measurement showed a significant correlation with age in the children. In adults, GFR/vECF significantly decreased with age; however, no significant association was shown between age and GFR/BSA [6]. Besides measurements of GFR and RPF, the cardiac output, Q(the volume of blood being pumped by the heart in the time interval of one minute), may change by body weight (per kg) or by body surface (cardiac index = Q/BSA) in children at different ages. The cytochrome P450 (CYP) enzymes constitute the major system for phase I metabolism, and in some CYP enzymes appear to be switched on by birth, while in others onset was later than birth [7,8]. However, for proteins and peptides, endopeptidase or receptor mediated transport processes are involved in hepatic metabolism instead of CYP enzyme [9,10]. The guidance for general industry considerations for pediatric pharmacokinetic studies for drugs and biological products by the FDA has summarized the effect of age and growth on absorption, distribution, metabolism and excretion (ADME) and protein binding in pediatrics [11]. The guidance has also pointed out that “In the pediatric population, growth and developmental changes in factors influencing ADME also lead to changes in pharmacokinetic measures and/or parameters. To achieve AUC and Cmax values in children similar to values associated with effectiveness and safety in adults, it may be important to evaluate the pharmacokinetics of a drug over the entire pediatric age range in which the drug will be used. Where growth and development are rapid, adjustment in dose within a single patient over time may be important to maintain a stable systemic exposure.”

**Table 1:** Changes in the total body water, extracellular fluid and intracellular fluid constitution of body weight with age.

Age	Total body water (%)	Extracellular fluid (%)	Intracellular fluid (%)
Fetus (<3 months)	90	65	25
Neonate (Premature)	85	50	35
Neonate (Full-term)	75	40	35
Infant (4–6 months)	60	23	37
Adolescent	60	20	40
Adult	60	20	40

**Table 2:** Tissue distribution comparison between newborn and adults (organ weight expressed as % of total body weight).

Organ	Newborn	Adults
Muscle	25	40
Skin	4	6
Heart	0.5	0.4
Liver	5	2
Kidney	1	0.5
Brain	2	2

**Table 3:** Renal function: changes in the glomerular filtration rate (GFR) and renal plasma flow (RPF) with age.

Age	GFR (mL/min)	RPF (mL/min)
1–10 days	15–45	20–125
1 month	30–60	100–400
6 months	50–100	400–500
1 years	80–120	500–600
1–70 years	80–140	500–700
70–80 years	70–110	250–450
80–90 years	45–85	200–400

### 1.2. Pharmacokinetics of Proteins and Peptides

Generally, pharmacokinetic principles are equally applied to the large molecule proteins and peptides and small conventional molecules. The underlying mechanisms for absorption, distribution, metabolism and excretion (ADME) of biologics are usually quite different from that of small molecules [12,13]. Therefore, in order to interpret and apply the pharmacokinetics of biologics, a thorough understating of ADME in these proteins and peptides is required. With few exceptions, almost all proteins and peptides are administered with intravenous, subcutaneous, or intramuscular dosage forms.

#### 1.2.1. Distribution

The volume of distribution of a molecule is affected by its physiochemical properties (such as lipophilicity and charge), protein binding, and possibly active transporters. Due to the large size of proteins and peptides, they usually exhibit small volumes of distribution, limited by the volume of the extracellular space, due to their mobility and inability to pass through membranes [14,15]. However, binding to intravascular/extravascular proteins or active tissue uptake can significantly increase the volume distribution of biologics [16]. The pharmacokinetics of proteins and peptides are usually described by a two compartment model, and volume of distribution of the central compartment as well as the steady-state volume of distribution (Vss) are usually reported [17]. The typical volume of distribution of the central compartment is equal or slightly bigger than the plasma volume of 3–8 L, and the Vss usually falls in the range of 14–20 L. It should be noted that the assumption to obtain Vss is not suitable for many biologics, since proteolysis in peripheral tissues may contribute significantly to the overall elimination process of these drugs [13]. Therefore, caution should be taken when interpreting Vss for proteins and peptides. Take antibodies for example: The Vss values reported in the literature for antibody pharmacokinetics studies are usually based on noncompartmental analysis, which assumes that the site of elimination reaches rapid equilibrium with plasma and that elimination is only through the central compartment. These assumptions do not necessarily apply to antibodies since antibodies also degrade in the tissues [13]. Protein binding has been reported to affect the transport and regulation of some proteins and peptides such as growth hormone, recombinant human growth factor, cytokines and fusion proteins (e.g. enfuvirtide) [18,19,20,21]. 

#### 1.2.2. Elimination

Peptides usually have short elimination half-lives, which is desirable for drugs like hormones, while large proteins like antibodies have an elimination half-life of around 21 days [13]. Biotechnological peptides and proteins are almost exclusively metabolized through the same catabolic pathways as dietary proteins and endogenous biologics. With few exceptions, renal or biliary excretions are generally negligible for most peptides and proteins. Proteases and peptidase are widely available throughout the body. Therefore, besides metabolism in the liver and kidneys, blood and other tissues are also sites of extensive metabolism for proteins and peptides. Renal elimination was reported for small proteins and peptides through predominantly three routes. Glomerular filtration of interleukin-11, growth hormone, and insulin was described previously [22,23,24]. Some of the small linear peptides are eliminated through hydrolysis by brush border enzymes on the luminal membrane, such as angoitensin I/II, glucagons and luteinizing hormone-releasing hormone [25,26,27]. Peritubular extraction of immunoreactive growth hormone and insulin has also been reported [22,28]. Several proteins and peptides were reported to be the substrates for hepatic metabolism including insulin, glucagon, epidermal growth factor, and antibodies [10,29]. Endopeptidase or receptor mediated transport processes were observed in the liver as well [9,10]. Many therapeutic proteins and peptides are endogenous molecules; receptor-mediated uptake followed by intracellular metabolism can take place in the organs that express receptors for these molecules. Since the number of receptors is limited, saturation can happen within the therapeutic concentration range. This saturation of the receptor-mediated elimination is a major source contributing to nonlinear pharmacokinetics of many proteins and peptides [30]. Nonlinear pharmacokinetics due to receptor-mediated drug disposition has been often reported for monoclonal antibodies [31,32]. 

In the current review, we summarize the effect of body size (body weight or body surface area) or age on pharmacokinetic parameters of selected biological products in pediatric patients such as clearance, volume of distribution, area under the curve (AUC), maximum concentration (Cmax), half-life, *etc*. Comparison of these parameters and the relationship of the parameters with body weight and age between pediatrics and adults are also included. The current review selectively includes the original clinical pharmacokinetic safety studies in pediatrics that have recorded body weight/BSA or age and/or incorporate body size and age in pharmacokinetic analysis. The dosing strategy of biologics in pediatrics is discussed accordingly.

## 2. Results and Discussion

An overview of the pharmacokinetics of selected FDA-approved proteins and peptides is presented in Table 4. Comparison of the pharmacokinetic parameters between pediatrics and adults, as well as the effect of body size and/or age on these parameters are discussed in the following section. 

### 2.1. Monoclonal Antibodies (mAbs)

#### 2.1.1. Basiliximab

In a pharmacokinetic and dosing rational study, 39 pediatric renal transplant patients were enrolled [33]. In part 1 of the study, pediatric patients were given 12 mg/m^2^ of basiliximab; in study part 2, infants and children received two 10 mg doses and adolescents received two 20 mg doses. Basiliximab clearance in infants and children (n = 25, 17 ± 6 mL/h) was reported to be approximately half that of adults (n = 169, 37 ± 15 mL/h) from a previous study [34], and was independent of age (1–11 years), body weight (9–37 kg), and body surface area (0.44–1.20 m^2^). Clearance in adolescents (12-16 years, n =14, 31 ± 19 mL/h) was comparable to the adult values. A similar designed study was carried out in liver transplant pediatric patients [35], and together, these data support a simple dosing algorithm for basiliximab in pediatric transplant patients. An adjusted fixed-dose of two × 10 mg is recommended for pediatric patients weighing less than 35 kg, and two doses of 20 mg is recommended for pediatric patients weighing 35 kg or more, just like for adults. 

In another study of basiliximab in pediatric renal recipients on comedication with mycophenolate mofetil, patients were classified by age as 16 children (3–11 years) and 27 adolescents (12–18 years) [36]. This study confirmed that the dosing strategy mentioned by the studies above provides consistent exposure for children and adolescents. Body surface area–adjusted basiliximab clearance was reported to be significantly higher in children. However, children were given a higher dose than adolescents (0.54 ± 0.18 *vs.* 0.42 ± 0.08 mg/kg). Similar total AUC were observed in the two groups (101 ± 68 µg d/mL in children *vs.* 102 ± 42 µg d/mL in adolescents), which resembled those of adults (107 ± 44 µg d/mL) from a previous study [37]. Significantly larger central and steady-state volumes of distribution were reported in children and adolescents than in adults, whereas half-lives were similar, 10.1 ± 7.6, 12.1 ± 5.0 and 11.5 ± 4.3 days in children, adolescents and adults, respectively. All the data implies that the body surface area adjusted dosing approach does not offer any apparent advantage over the simpler fixed-dose approach.

**Table 4:** Pharmacokinetics of selected proteins and peptides in pediatrics.

Generic Name	Class	Route	Pharmacokinetics
Alemtuzumab	mAbs	i.v.	More rapid clearance in children than in adults.
Basiliximab	mAbs	i.v.	CL (mL/h) in infants and children is about half that of adults. Use 35 kg as a cut-off weight for 10 or 20 mg in pediatrics.
Bevacizumab	mAbs	i.v.	Body weight (BW) based dose exhibits similar PK parameters in children and adults, and large variability in both populations.
Cetuximab	mAbs	i.v.	Dose-dependent nonlinear elimination. BSA based dose provides similar exposure in children and adults, and age has no effect on PK.
Daclizumab	mAbs	i.v.	The 4.2-fold range in CL, 7.4-fold range in V are less proportional than a 12-fold range in body weight
Darbepoetin Alfa	Growth factor	i.v., s.c.	The lack of dose-proportionality is likely due to pediatric population rather than nonlinear PK; neonates have a shorter half-life, larger V and CL than children.
Drotrecogin alfa	Blood factor	i.v.	Weight-normalized clearance decreases significantly with age in patients <18 years old.
Enfuvirtide	Peptide	s.c.	One study justified body weight (BW) based pediatric dosing.
Epoetin Alfa	Growth factor	i.v., s.c.	CL (mL/h/kg) and bioavailability in pediatrics were two-fold of that in adults.
Epoetin Delta	Growth factor	i.v., s.c.	BW adjusted PK parameters are similar in children and in adults.
Etanercept	Fusion protein	s.c.	The analysis justified the body weight based dose adjustment for etanercept in JRA patients; gender difference was reported both in children and adults.
Exenatide	Incretin	s.c.	The max recommended adult dose instead of half of the max dose was suggested to be explored in adolescent patients.
Factor VII	Blood factor	i.v.	Total body weight normalized clearance was significantly faster in children than in adults.
Factor VIII	Blood factor	i.v.	BW adjusted clearance in mL/h/ kg and Vss in L/ kg seems to decrease with age.
Factor VIX	Blood factor	i.v.	Higher weight-adjusted CL in children than adults.
Filgrastim	Growth factor	s.c.	ANC-adjusted G-CSF dosing adjustment might improve PBPC mobilization in pediatric patients.
Gemtuzumab	mAbs	i.v.	Both faster CL (L/h) and CL (L/h/m^2^) in adults than children and infants.
Humatrope	Growth hormone	s.c.	No significant effect of weight and age on humatrope pharmacokinetic parameters.
Infliximab	mAbs	i.v.	BW based dose provides similar exposure in children and adults; PK of infliximab does not differ as age increases.
Insulin aspart	Insulin	s.c.	In pediatrics, insulin aspart had a quicker onset than human insulin; aspart has a higher exposure in adolescents than in children.
Insulin detemir	Insulin	i.v.	Less PK variability in insulin determir than glargine.
Insulin glulisin	Insulin	i.v.	The profile of insulin glulisine is similar for children and adolescents, whereas human insulin exhibits higher level in adolescents.
Interferon-α2a	Interferon	s.c.	Higher drug exposure in pediatrics; wide intersubject variability suggests further individualized dosing.
Interferon-α2b	Interferon	i.v.	BSA based PK parameters in pediatrics is about twice that in adults.
Interferon-αnl	Interferon	i.v., i.m.	No BW/BSA or age affect was discussed. Slightly lower exposure in pediatrics than in adults.
Interleukin	Cytokines	i.v., s.c.	Higher rhIL-11 clearance in pediatrics than adults
Asparaginase	Enzyme	i.m, i.p.	After adjusting dose by BSA, neither age nor the BSA had any influence on the distribution.
LB03002	Growth hormone	s.c.	Body weight adjusted dosing gives comparable exposure in pediatrics to in adults.
Natalizumab	mAbs	i.v.	BW base dose tends to underdose adolescents.
Nutropin	Growth hormone	s.c.	Drug exposure was approximately proportional to the dose.
Palivizumab	mAbs	i.v., i.m.	BW based dose for palivizumab, but body weight effect not discussed; no significant clinical outcome between placebo, 5 and 15 mg/kg were observed.
Somatropin	Growth hormone	Inhaled	No significant effect of weight and age on somatropin pharmacokinetic parameters.
Zomacton	Growth hormone	s.c.	No BW/BSA or age correlation was analyzed for its pharmacokinetic parameters.

i.v.: intravenous; s.c.: subcutaneous; mAbs: monoclonal antibodies; CL: clearance, PK: pharmacokinetics, BSA: body surface area, V: Volume of distribution, JRA: juvenile rheumatoid arthritis, ANC: absolute neutrophil count, G-CSF: granulocytecolony simulating factor, PBPC: peripheral blood progenitor cell, rhIL-11: interleukin-11.

#### 2.1.2. Daclizumab

In a study of daclizumab, pediatric renal transplant recipients were divided into different age groups (≤ 5 years (n = 18), 6–12 years (n = 18), and 13–17 years (n = 25)), and the analysis indicated that bodyweight and race (black *vs.* nonblack) were found to be significant influences on the pharmacokinetics of daclizumab in pediatric patients [38]. A 4.2-fold range in Clearance (CL) (4.50–19.0 mL/h) and a 7.4-fold range in central volume of distribution (V1: 0.64–4.71 L) were less proportional than a 12-fold range of bodyweight (7.5–89.5 kg). As a result, body weight adjusted dosing leads to lower exposure in the younger patient group (<5 years), and higher exposure in patients with larger body weight. The pharmadynamic results showed that the difference in exposure did not affect the safety and extent of daclizumab saturation in different age groups.

#### 2.1.3. Palivizumab

The intramuscular humanized monoclonal antibody palivizumab was studied in premature infants and infants with bronchopulmonary dysplasia using body weight adjusted dosing [39]. Sixty-five infants (ages 4.6 to 7.6 months) were enrolled of whom 11 (17%) received 5 mg/kg, six (9%) received 10 mg/kg and 48 (74%) received 15 mg/kg palivizumab. Mean serum palivizumab concentrations (ranges) measured at two days were 28.4 µg/mL (13.0–41.1) and 91.1 µg/mL (52.3–174.0) for the 5 mg/kg and 15 mg/kg dose groups, respectively, and after 30 days the palivizumab levels were 12.5 (4.2 to 26.2) and 49.2 (13.5 to 132.0) µg/mL. The study concluded that monthly injections of 15 mg/kg were able to maintain mean serum concentrations above 40 µg/mL. The safety and pharmacokinetics of palivizumab was studied in 59 children ≤2 years with respiratory syncytial virus infection [40]. Mean palivizumab levels were 61.2 and 303.4 µg/mL at 60 min after infusion and 11.2 and 38.4 µg/mL at 30 days after infusion of 5 and 15 mg/kg palivizumab, respectively. The mean half-lives were 22.6 and 16.8 days after the infusion of 5 and 15 mg/kg palivizumab, respectively. The mean area under the curve was 487 µg/mL after 5 mg/kg and 2386 µg/mL after 15 mg/kg. No significant differences in clinical outcomes between placebo and 5 or 15 mg/kg palivizumab were observed.

#### 2.1.4. Infliximab

Infliximab was studied for the first time in a clinical trial in patients younger than 12 months (six infants and 10 children) [41].The pharmacokinetics of infliximab (5 mg/kg) did not differ as age increased. Standard body weight adjusted dosing provided peak concentrations similar to those reported previously, regardless of subject age. The peak concentrations were similar to those observed in a study with peak and trough levels reported after a dose of 6 mg/kg in 62 children (ages 4 to 17 years) with pauciarticular juvenile rheumatoid arthritis [42]. The single dose of 5 mg/kg used in the study with infants and children exhibited comparable systemic infliximab exposure to that reported previously for therapeutic drug monitoring of infliximab in adolescents and adults [43]. The estimated pharmacokinetic parameters median (CV) in the five pediatric patients were volume of distribution (V) 3.0 L (13%), clearance (CL) 0.0083 L/h (40%), and half-life (t1/2) 10.9 days (20%) [43]. The parameters are consistent with a study by Cornillie that reported a median t1/2 of 9.5 days and a median CL of 0.0098 L/h [44], and another study that reported a t1/2 of 8–12 days (n = 108, 1, 5, 10, 20 mg/kg) [45]. In yet another study, 21 pediatrics, aged 8–17 years, were given an infliximab dose of 1, 5, or 10 mg/kg. Serum infliximab concentrations were reported to be proportional to the dose, and the pharmacokinetic profile of the pediatric patients was similar to that of adults [46].

#### 2.1.5. Gemtuzumab

Gemtuzumab is derived from the murine anti-CD33 antibody hP67.6. In a pediatric pharmacokinetic study of gentuzumab, twenty-nine patients were grouped into three age categories: infants (0–2 years), children (3–11 years), and adolescents (12–16 years) [47]. Dosages of 6, 7.5, and 9 mg/m^2^ gentuzumab were given to the pediatric patients. Pharmacokinetic parameters of hP67.6 antibody for the first dose are consistent and statistically different from that of the second dose. Increases in AUC and decreases in CL and Vss from the first dose to the second dose in pediatrics agree with those reported for the adults. Reported mean pharmacokinetic parameters in pediatrics are similar to the values reported in adults [48]. Children given the dose of 9 mg/m^2^ had the hP67.6 parameters of: Cmax, 3.47 ± 1.04 mg/L; AUC, 136 ± 107 mg·h/L; CL, 0.12 ± 0.15 L/h/m^2^; Vss, 6.5 ± 5.5 L; and t1/2, 64 ± 44 h after the first dose. Concentration *vs.* time profiles of hP67.6 was similar for the first dose among age. The mean Cmax for infants was a bit lower than for children, and the Cmax for children was 22.8% higher than for the adolescents. The AUC of infants and children were 2.3% and 33.5% higher than adolescents. CL in infants and children was 80.9% and 72.0% lower, respectively, than in the adolescents. The CL (L/h) values after administration of 9 mg/m^2^ gentuzumab in infants, children, adolescents and adults were 0.03 ± 0.02, 0.06 ± 0.03, 0.26 ± 0.30 and 0.27 ± 0.23, respectively. The body surface area adjusted CL (L/h/m^2^) values in infants, children, adolescents and adults were 0.05 ± 0.02, 0.05 ± 0.05, 0.17 ± 0.21 and 0.15 ± 0.13, respectively. Therefore, both absolute CL (L/h) and body surface area adjusted CL (L/h/m^2^) increase from infants to adult age. Volume of distribution showed the same trend: values were lower in infants and children than in adolescents. Body weight adjusted Vss (L/kg) was larger in adults and infants than in children and adolescents. There was no statistically significant correlation observed between hP67.6 CL and body weight or CL and age. Intersubject variability within age groups was relatively large for the pharmacokinetic parameters. Overall, the body surface area adjusted dose provides comparable exposure for pediatric patients.

#### 2.1.6. Alemtuzumab (Campath-1H)

In a phase II study, Campath-1H 0.6 mg/kg (max 30 mg) was administered in 13 (eight male) pediatric patients with a median (range) age of eight (3–20) years [49]. The study concluded that Campath-1H exposure in pediatrics with acute lymphoblastic leukemia (ALL) tends to be lower than that in adults with chronic lymphocytic leukemia (CLL) [50], and this observation may be due to the more rapid clearance in children. This indicated that children may have higher body weight normalized clearance than adults. Mould *et al*. reported that adult patients with a Campath-1H trough concentrations >13.2 mg/mL had a 50% chance of achieving either complete remission or partial remission [50], while Montillo *et al*. reported that all patients with a Campath-1H AUC0–12 >5 mg hr/mL achieved a complete remission [51]. In a study of 30 CLL patients, mean peak and trough plasma concentration was 10.7 mg/mL (2.8–26.4 mg/mL) and 5.4 mg/mL (0.5 to 18.3 mg/mL). It was found that not all patients showed beneficial clinical response, and higher blood peak concentrations correlated with better clinical outcome [52]. 

#### 2.1.7. Cetuximab

In a phase I study, 27 children (aged 1–12 years) and 19 adolescents (aged 13–18 years) received escalating weekly doses of cetuximab (75, 150, 250 mg/m^2^) [53]. In the dose range studied, cetuximab exhibited nonlinear pharmacokinetics, since the AUC did not increase proportionally as the dose increased. The clearance after non-compartmental analysis decreased with increasing dose in both children and adolescents. In children, clearance decreased from 0.057 to 0.015 L/h·m^2^ as cetuximab dose increased from 75 to 250 mg/m^2^. Similar results were reported in adolescents. The receptor-mediated clearance might explain this dose-dependent elimination of cetuximab, and the receptors are likely to be saturated at higher doses. The mean steady-state volume of distribution across all doses and age groups was around 2 L/m^2^, indicating limited distribution of cetuximab into the extracellular space. Overall, cetuximab exhibits nonlinear pharmacokinetics and similar profiles among age groups. Estimates of the pharmacokinetic parameters (clearance, area under the curve, and volume of distribution) at steady state in both the children and adolescent subgroups were comparable to those previously reported in adults [54]. The body surface area adjusted dosing seems to provide consistent exposure in children and adolescents compared to that of adults, and the pharmacokinetics does not seem to correlate with age in pediatric patients. 

#### 2.1.8. Bevacizumab

In a phase I study of 20 pediatric cancer patients, aged 1 to 20 years (median 13 years), 10 females and 10 males, bevacizumab exposure was proportional to dose (5, 10, 15 mg/kg) [55]. The study showed a large degree of interpatient variability in children, which was similar to that observed in adults [56]. Bevacizumab exhibits linear pharmacokinetics at the dose range of 1 to 20 mg/kg in adults [56]. Median clearance and mean residence time in children and adults were 4.1 *vs.* 3.9 mL/d/kg and 16.3 *vs.* 12.4 days, respectively [55,56]. In a population pharmacokinetic study of bevacizumab, gender difference was found in adult patients [57], but with a limited number of pediatric patients, the gender analysis was not performed in the study [50]. 

#### 2.1.9. Natalizumab

In a pediatric study, 38 adolescent patients (aged 12–17 years) with active pediatric Crohn Disease received three intravenous infusions of natalizumab (3 mg/kg) at 0, 4 and 8 weeks [58]. The natalizumab peak level and half-life after the first and third infusions were 61.0 *vs.* 66.3 mg/mL and 92.3 *vs.* 96.3 h. Natalizumab showed time-invariant pharmacokinetics and no accumulation on repeated monthly dosing. The Cmax and half-life of natalizumab (3 mg/kg) in the adolescents were reported to be lower and shorter compared with those in adults after a fixed dose of 300 mg. The study showed that the dose of 3 mg/kg in adolescent patients may reduce the symptoms of severe or moderate Crohn Disease. Overall, the study concluded that the magnitude of the clinical benefit to adolescent patients is unknown, because the body weight based dosing of 3 mg/kg did not provide adequate receptor saturation in adolescents. This study is an example where simple body weight adjustment for dose in adolescents has the potential to underdose the population. 

### 2.2. Growth Factors

#### 2.2.1. Epoetin Alfa and Delta

In a pharmacokinetic and pharmacodynamic study, of 12 children enrolled with cancer, six (median age 15.2 years; range 9.3–18.6 years) were randomized to receive erythropoietin (EPO) [59]. In this study, children were randomized to receive i.v. EPO 600 IU/kg (max dose 40,000 IU) or placebo weekly for 16 weeks. Doses for all the children were increased to 900 IU/kg (max dose 60,000 IU) since a 1 g/dL increase in hemoglobin was not observed by study week 3 or 4. EPO clearance after the first dose showed relatively big intersubject variability (0.19–1.08 L/h/m^2^), but the clearance after the 10th and 11th dose showed much less intersubject variability (0.15 to 0.25 L/h/m^2^). Additionally, the AUC_0–24_ of EPO increased proportionally with EPO dose in these children. 

In a previous study in adults, the mean half-life and clearance after the first EPO dose were 7.7 h (range 3.5–12.6 h) and 0.4 L/h (range 0.3–0.7 L/h), respectively [60]. If adjusting for BSA, the study exhibits similar pharmacokinetic parameters as the above study in children with cancer. For example, an adult with a typical BSA of 1.73 m^2^ has clearance of 0.4 L/h, which equates to 0.2 L/h/m^2^. Another intravenous EPO (40 IU/kg) study in children (aged 9–16 years) reported a mean half-life and clearance of 5.6 h (range 4.4–6.7 h) and 10.1 mL/h/kg (range 7.1–14.9 mL/h/kg) [61]. The study also concluded that after i.v. administration, clearance in pediatrics was two-fold that in adults, and after s.c. dosing, bioavailability was two-fold of that in adults. This study showed similar clearance to the cancer children study when adjusting the clearance in cancer children for body weight (12.4 mL/h/kg). Unlike the above studies in children, premature infants (birth weight < 1.25 kg) showed greater serum erythropoietin clearance and larger volume of distribution than adults [62]. Two more studies have reported greater clearance in pre-term infants than adults after continuous intravenous or multiple subcutaneous EPO administration, and larger bioavailability was reported in pre-term infants than adults given subcutaneous EPO [62,63].

A population pharmacokinetics study of intravenous and subcutaneous epoetin delta in pediatric patients with chronic kidney disease discussed the covariate effects on epoetin delta and epoetin alfa pharmacokinetic parameters [64]. Of 60 patients, 47 of them received i.v. or s.c epoetin delta and 13 of them received i.v. or s.c. epoetin alfa. In the population pharmacokinetic modeling building, V and CL were allometrically scaled by body weight by fixing the power exponents to 0.75 for CL and 1 for V. Age was included in the final model by a power function, normalized by the reference age of 10 years for children older than 10; sex, dialysis type, and drug type were also included in the model. The typical pharmacokinetic estimates were CL (0.268 L/h), V (1.03 L), Ka (0.0554 h^–1^), and bioavailability (0.708) for a 35 kg male ≤ 10 years who was given s.c. epoetin delta and on predialysis. The epoetin delta pharmacokinetic parameters were similar in children as compared with those in adults when normalized by weight [65]. The subcutaneous epoetin alfa reported lower bioavailability than subcutaneous epoetin delta.

#### 2.2.2. Darbepoetin Alfa

This was a randomized, open-label, crossover study in pediatric patients with chronic kidney disease (CKD), with a mean age of 11 (range 3–16 years) [66]. Twelve patients with CKD were randomized to receive a single dose of 0.5 μg/kg i.v. or s.c darbepoetin alfa. The mean clearance and half-life of darbepoetin alfa was 2.3 mL/h/ kg and 22.1 h after i.v. administration. Absorption was shown to be the rate limiting step after s.c. darbepoetin alfa; the mean half-life was 42.8 h and mean bioavailability was 54%. Beside slightly faster absorption for s.c. administration, darbepoetin alfa disposition in pediatrics were shown to be similar to that in adults patients [67]. Darbepoetin alfa exhibited roughly two- to four-fold longer terminal half-life than previously reported in epoetin in pediatric patients [61]. Previous studies in adult patients with CKD showed that the darbepoetin alfa half-life was approximately three-fold longer than that of i.v. epoetin (25.3 h *vs.* 8.5 h) and around two -fold longer than s.c. epoetin (48.4 h *vs*. 24 h) [67,68,69]. In another study in pediatric patients with chemotherapy-induced anemia (CIA), 16 patients (mean age 12 years, range 5–18 years) were given darbepoetin alfa 2.25 μg/kg subcutaneously [70]. After a single dose of s.c. darbepoetin alfa, the mean (SD) terminal half-life of 49.4 (32) h was found to be similar to the 48.2 h in pediatric CKD patients [66]. The lack of dose-proportionality in the Cmax between the 0.5 μg/kg in the CKD patients and 2.25 μg/kg in the CIA patients is likely due to population differences rather than nonlinear pharmacokinetics. Darbepoetin alfa showed linear pharmacokinetics in adults patients. 

In a study in neonates, a single i.v. dose (4 mg/kg) of darbepoetin was given to 10 neonates who had a hemoglobin ≤ 10.5 g/dL. The birth weight of the neonates was 1,128 g (median, ranged from 704 to 3,025 g), and were 26.0–40.0 weeks old (median, 29.2 weeks). The mean (range) half-life, V and CL in the pre-term neonates were 10.1 h (range 9.0–22.7 h), 0.77 L/kg (range 0.18–3.05 L/kg), and 52.8 mL/h/kg (range 22.4–158.0 mL/h/kg) respectively. In preterm neonates, there was no significant correlation between age and darbepoetin pharmacokinetic parameters. V was found to be correlated with both age and gestational age in the term and near-term neonates. Darbepoetin i.v. pharmacokinetics in neonates was compared with children, and neonates had a shorter half-life, a larger V and larger CL than children [71].

#### 2.2.3. Filgrastim

A different dosage adjustment, other than body weight or age based dose, was used for granulocyte-colony stimulating factor (G-CSF) in pediatric patients, aged two to 17 years [72]. Because G-CSF clearance increases with increasing absolute neutrophil count (ANC), the dose optimizing study of G-CSF was conducted by giving eight patients filgrastim at a single dose of 10 mg/kg/day subcutaneously for peripheral blood progenitor cell (PBPC) mobilization. This preliminary pharmacokinetics of G-CSF seems to indicate that an ANC-adjusted G-CSF dosing adjustment might improve PBPC mobilization in pediatric patients.

### 2.3. Interferon

In a phase I pharmacokinetic study of interferon-αnl (IFN-αnl), 12 children, aged 3–15 years, with relapsed acute lymphocyte leukemia (ALL) were given IFN-αnl intravenously or intramuscularly for over 25 days [73].Single doses of 2.5 to 15 MU/m^2^ (total doses of 60 to 200 MU/m^2^) were given to the subjects. The serum levels of IFN and the AUC were similar to those reported in adult cancer patients, but slightly lower [74]. The study did not discuss the body surface area, body weight or age effect on pharmacokinetic parameters. The individual AUC was reported, but due to the unknown information of the total dose, the relationship of age or body size with dose adjusted AUC could not be evaluated. 

A safety and pharmacokinetic study of PEG-interferon alpha2a was done in 14 children, aged 2–8 years (mean age 4.4 years) with chronic hepatitis virus infection (HCV) [75]. Mean (range) weight was 20.1 kg (13.3–45.3 kg). The drug dose was calculated based on the patients’ body surface area (BSA) using the formula BSA (m^2^)/(1.73 m^2^) × 180 μg. BSA was found as a linear covariate for apparent clearance and body weight was found to be a linear covariate for apparent volume of distribution of the central compartment. The study showed wide intersubject variability with the apparent clearance range of 6.6–35.5 mL/h in the 14 pediatric patients, which suggested the necessity of individualized dosing. When compared with data from a phase III 48-week adult study, the mean Ctrough in children was comparable to that in adults [76]. The mean AUC_0-168h_ was 25% higher in pediatrics than in adult. Standard interferon (INF) has shown better efficacy in pediatric patients; additionally, children seem to tolerate pegylated INF better than adults. As a result, the study concluded that the higher drug exposure in pediatric patients may have potentially good efficacy outcomes.

Among the 56 pediatric patients (ages 3–16 years) who participated in the multiple-dose pharmacokinetic interferon alfa-2b study, 20, 19, and 17 subjects received 8, 12, and 15 mg/kg/d of ribavirin, respectively [77]. Median (range) body weight of the subjects was 40.4 kg (10–95). The pharmacokinetics of interferon alfa-2b in children was approximately twice that of adults on a body surface area basis. The dose normalized AUC_0-12h_ and Cmax were similar to the multiple-dose pharmacokinetics in adults.

### 2.4. Blood Factors

#### 2.4.1. Factor VII

Pharmacokinetics of activated recombinant coagulation factor VII (NovoSeven) was compared in children *vs.* adults with hemophilia A [78]. Twelve children (2–12 years) received rFVIIa at one single dose of 90 and 180 µg/kg. In children, the plasma FVII concentration was dose proportional in the dose range of 90–180 µg/kg. Direct comparison of the results for adults (ages 18–55 years) and children (2–12 years) reflects that plasma clearance was significantly higher in pediatrics than in adults for both the FVII:C and FVIIa clot activity assays. The total body weight normalized clearance was significantly faster in children than in adults with both assays (rFVII:C, 58 *vs.* 39 mL/kg/ h and rFVIIa, 78 *vs.* 53 mL/kg/ h). This difference suggests a higher metabolic activity per kg body weight in children than in adults and is likely correlated with age-related differences in body composition, such as different liver volume per kg body weight, as previously described [79]. This difference also suggested a higher dose of rFVII might be needed for children to achieve the comparable levels to in adults. The relationship between clearance and weight was illustrated by a linear regression in a review as CL (mL/kg/h) = 76.8−0.488 × (Weight−43.6 kg) (p < 0.002) [80]. Volume of distribution at steady state tends to be larger in children than in adults, but not significantly (196 *vs.* 159 mL/kg). The dose-normalized AUC0-12 was 30% lower in children than in adults. This study is important for pediatric dosing of FVII as it provides predictable pharmacokinetics in children.

#### 2.4.2. Factor VIII

A study for the first time analyzed the effect of age and body mass index (BMI) on pharmacokinetic parameters in young children for prediction of dosage regimen [81]. Pediatric patients (52 boys, one girl, mean ± SD age 3.1 ± 1.5 years) were given an intravenous bolus dose of rAHF-PFM (recombinant anti-hemophilic factor-protein-free method) of 50 IU/ kg. BMI was a significant predictor of Factor VIII distribution. Vss decreased linearly as BMI increased, and age was a significant covariate for half-life and MRT. In another study of rAHF-PFM with 111 subjects, with a median age of 18, rAHF-PFM mean (±SD) half-life was 12.0 ± 4.3 h [82]. A study in a premature infant showed the half-life of Factor VIII was 6.43 h, and 6–20 h in children from other studies [83]. 

Twenty-one patients (aged 8–42 years), including 12 pediatric patients, received single doses of 24–51 U/ kg [84]. The pharmacokinetic parameters were CL: 81–606 mL/h, V: 1.6–9.7 L, half-life: 7.8 to 18.3 h. Weight was found to correlate with clearance and Vss, and a positive correlation of age and half-life was reported. It was shown that when body weight increased from 40 to 80 kg, this 100% increase in body weight corresponded to an increase of 42% in clearance and an increase of 60% in Vss. Clearly, this increase of clearance and Vss is not proportional to the weight change. Normalization of clearance and Vss for total body weight will therefore not correctly explain the interindividual differences but rather over-correct them. Additionally, body weight adjusted clearance in mL/h/ kg and Vss in L/ kg seems to decrease with age. The half-life of FVIII tended to be shorter in pediatrics than in adults.

In a retrospective study, patients 7–77 years old (one child, 16 teenagers, and 44 adults; body weight 21 to 120 kg) were given FVIII to determine the pharmacokinetics of FVIII [85]. The body weight normalized pharmacokinetic parameters of pediatrics were comparable with those observed in adults. Covariate analysis showed that V1 is significantly related to body weight and BSA. Including BSA in the model substantially decreased the unexplained V1 variability (from 34.1% to 21.1%).

In a study of factor VIII (FVIII), 34 patients (aged 7–74 years), including 16 children, were used for model building [86]. Body weight and age were found to be significant covariates. FVIII was administered at around 60 U/kg in the small children, decreasing to 10 U/kg or less in middle age patients. The dose requirements after obtaining individual PK data showed a much greater variation than the dose range used. Weight normalized clearance (CL/kg) of FVIII has been reported to decrease with age and/or body weight during growth from infancy to adulthood, and half-life showed the opposite trend [84,87,88]. Pharmacokinetics of FVIII was well described by a two-compartment model. In the model building process, the exponent on clearance and volume of distribution was set to 0.75 for the clearance parameters (CL and Q) and to 1 for the volume (V1 and V2) terms. In addition to the influence of body weight on clearance, age showed a significant effect only on weight-adjusted CL, which decreased by 1.5 mL/h per year of age with a reference age of 24. Age showed no significant correlation with weight-adjusted V1, which was in line with a previous observation that age was not correlated with *in vivo* recovery (Cmax divided by dose) [89]. The study concluded that the right dosage of FVIII cannot be only calculated from body weight and/or age, and suggested that starting doses for most patients should be 1,000 U every other day. Individual FVIII concentrations should then be checked for further dose adjustment. 

#### 2.4.3. Factor IX

A six-year follow-up study was done for coagulation factor, Factor VIII and Factor XI (FIX), in children and adults with hemophilia [90]. The median CL of FVIII:C was 3.0 (range, 1.1–9.9) mL/h/kg, the Vss was 0.050 (0.028–0.129) L/kg, and the half-life was 11 (5.1–33) h. Clearance increased with increasing body weight in this patient population. A 100% increase in weight, from 40 to 80 kg, corresponds with a 39% increase in CL for FIX. The median CL of FIX:C was 3.9 (2.9–4.5) mL/h/ kg, the Vss was 0.14 (0.08–0.20) L/kg, and the half-life was 32 (26–49) h. The prophylactic dose of coagulation factor, in U/kg, was higher for children, especially small children, because of the higher weight-adjusted CL in children than adults.

Pharmacokinetics of Factor IX was studied in 56 patients, aged 4–56 years [91]. FIX:C clearance and volume of distribution at steady state increased linearly with body weight, with a faster increase in children and adolescents but remaining relatively constant during adulthood. The body weight adjusted CL and Vss, shown as functions of age, indicated a decrease of 0.68% of CL/body weight per year, and CL/lean body mass decreased by 0.40% per year. The slope between the two regressions was not statistically different, which indicates that dose adjustment of rFIX (recombinant FIX) to lean body mass did not reduce this variability compared to body weight dose adjustment. Vss/body weight decreased by 0.68% per year, while Vss/lean body mass decreased by 0.38% per year, and were not statistically different. The terminal half-life of FIX:C exhibited no correlation with age, nor MRT. The high intersubject variation in disposition and required doses of rFIX suggests the need for individual dose titration. 

#### 2.4.4. Drotrecogin Alfa

In the first study reporting the use of drotrecogin alfa (activated) in pediatric patients, the overall mean weight adjusted clearance was 0.53 L/h/kg across all infusion rates and age groups (n = 63). No correlation was found between infusion rate and age group. Weight-normalized clearance decreased significantly with age in patients <18 years old, although combined pediatric and adult weight-normalized clearance was not found to depend significantly on age or body weight. The mean weight-normalized CL in patients <3 months (n = 11) (0.608 L/h/kg) was 22% higher than that in all patients three months or older (0.497 L/h/kg) and 19% higher than that in adult patients (18 years or older). The higher CL in the small children was expected to have slightly lower steady-state concentration than in the older patients.

### 2.5. Hormones

#### 2.5.1. Insulin Hormones

A trial enrolled 32 children and adolescents (19 girls and 13 boys; aged 13 ± 2.5 years, range 6–17 years) to compare the pharmacokinetics of detemir and glargine [92]. BMI was 15–24 kg/m^2^ for children ages 6–12 years and 18–29 kg/m^2^ for adolescents ages 13–17 years, but the study did not mention weight or age effect on variability of the two drugs. Pediatric patients were randomized to receive a sequence of 0.4 U/kg of detemir and glargine. The study concluded that the intersubject variability in pharmacokinetics was significantly lower for detemir than for glargine in type 1 diabetes mellitus (T1DM) children and adolescent patients. The smaller pharmacokinetic variability observed was most likely due to the smaller variability in absorption with detemir, which is also likely to be associated with a more predictable therapeutic range. 

An insulin comparison study in pediatrics reported that insulin aspart had a quicker onset and shorter duration of action compared with human insulin, meaning that aspart is more appropriate to inject immediately before a meal, which makes it a more practical product [93]. In this study, postprandial plasma glucose increments did not differ between the human insulin and insulin aspart. Slightly higher blood glucose concentration was observed after breakfast and dinner with insulin aspart administration. In another study, subcutaneous insulin aspart or human insulin (0.15 IU/kg body weight) was given 5 min before breakfast in nine children (aged 6–12 years) and nine adolescents (aged 13–17 years) with T1DM [94]. Insulin aspart exhibited significantly higher Cmax ± SD than human insulin (881 ± 321 pmol/L *vs.* 422 ± 193 pmol/L, p < 0.001). Cmax and AUC of insulin were found to be related with age in the study. The change of glucose AUC and Cmax were smaller for insulin aspart than human insulin in children. It was surprising for the investigators to find higher levels of both insulin aspart and human insulin in the adolescents than in children. Additionally, the insulin dosage in this study does not reflect the usual dosage of insulin in adolescents (1.0 ± 1.5 U/kg per 24 h) and smaller children or adults (0.5 ± 1.0 U/kg per 24 h). There is not an extensive comparison of pharmacokinetic study in children and adults in the literature. Pharmacokinetic study was done to compare insulin glulisine and regular human insulin analogous in children and adolescents with T1DM [95]. Ten children (aged 5–11 years) and 10 adolescents (aged 12–17 years) were enrolled. The concentration time profile for insulin glulisine was similar for children and adolescents, whereas human insulin exhibited 64% higher concentration in adolescents. The higher concentration of human insulin observed in adolescents is in line with the previous study [94]. The difference is suggested to be caused by disparities in residual endogenous insulin secretion in adolescents and children or simply the fact that adolescents were given a larger meal than the children.

Exenatide (5 and 10 μg Twice daily (BID)) was approved as an incretin mimetic in adults with T2DM. Thirteen adolescent patients (aged 10–16 years; seven females, six males; body mass index of 32.5 ± 5.0 kg/m^2^) were given 2.5 μg exenatide, 5 μg exenatide, or placebo followed by a standardized meal 15 minutes later [96]. There was no demographic effect, such as age, sex, race, or degree of obesity, found on exenatide pharmacokinetics in adults during clinical development. The exenatide AUC was found to be dose proportional in these adolescent patients. Postprandial plasma glucose levels were significantly decreased with both doses of exenatide compared with the placebo from one to three hours after administration. The geometric mean ± SE exenatide AUC_0–∞_ and Cmax were 339.5 ± 39.6 pg·h/mL and 85.1 ± 11.5 pg/mL after 5 μg exenatide (n = 12) and 159.2 (23.1) pg·h/mL (n = 6) and 56.3 (10.1) pg/mL (n = 9) after 2.5 μg exenatide. Not all exenatide levels were detectable in patients who received 2.5 μg exenatide. After 5 μg of exenatide was given to these patients, the geometric mean ± SE AUC_0–360min_ (195.9 ± 25.5 pg·h/mL) and geometric mean Cmax (85.1 ± 11.5 pg/mL) were comparable to those of adults with T2DM (n = 39) (AUC_0–360min_, 232.2 ± 30.3 pg·h/mL and Cmax, 113.0 ± 12.2 pg/mL) [97,98,99]. With this finding, the study suggested that the recommended adult dose of 5 μg and the maximal recommended adult dose of 10 μg should be explored in adolescent patients.

#### 2.5.2. Growth Hormone

Somatropin inhalation powder and subcutaneous humatrope pharmacokinetics were compared in pediatrics with growth hormone deficiency (aged 6–16 years, weighing 18.0–52.0 kg, with a mean BMI of 17.5 kg/m^2^ [100]. Participants were randomized to one of three dose levels: 1) 8.4 mg/d somatropin or 0.5 mg/d humatrope; 2) 16.8 mg/d somatropin or 1.0 mg/d humatrope; 3) 33.6 mg/d somatropin or 2.0 mg/d humatrope. At least two subjects were assigned to each dose level within each of the weight ranges: 18.0–29.9, 30.0–39.9, and 40.0–52.0 kg. The mean serum growth hormone area under the curve of somatropin was dose proportional. There was no significant effect of weight and age on somatropin and humatrope pharmacokinetic parameters. Height was found to be a significant covariate for somatropin AUC, somatropin Cmax, and humatrope AUC, respectively, which indicates taller subjects tended to have higher AUC and Cmax.

A novel sustained-release recombinant human growth hormone, LB03002, given by s.c. injection once-a-week, was studied in 37 children (24 boys, 13 girls, ages 6.5 ± 2.1 years), at doses of 0.2, 0.5 or 0.7 mg/kg [101]. Cmax and AUC was dose proportional in the dose range of 0.2–0.7 mg/kg, and was comparable with the levels in adults [102]. This study shows that body weight adjusted dosing of LB03002 gives comparable exposure in pediatrics as in adults.

Nutropin Depot was administered subcutaneously in 138 pediatrics, and the Cmax and total growth hormone (AUC_0–28 d_) were approximately proportional to the dose administered (0.75 mg/kg twice a month and 1.5 mg/kg once a month) [103]. 

Zomacton 2 IU/m^2^ jet-injected and needle-injected was studied in 18 pediatrics, and the AUC, Cmax and Tmax were similar for both groups [104]. The study reported the individual BMI, age and sex information for the subjects, but due to the limited number of patients, no correlation was demonstrated with the pharmacokinetic parameters.

### 2.6. Other Proteins and Peptides

#### 2.6.1. Interleukin

In a dose escalation study in children, adolescents and young adults of recombinant human interleukin-11 (rhIL-11), Cmax and AUC were dose proportional, with a mean Cmax level (range, 7.6–25.5 ng/mL) and AUC (range, 56.7–208.6 ng·h/mL) at a dose range of 25–100 μg/kg [105]. The pharmacokinetics of intravenous and subcutaneous rhIL-11 at a dose range of 3–50 μg/kg was studied in 30 healthy male adults [106]. The adult mean Cmax and AUC was reported to be dose proportional, which is similar to the pediatric study. At their overlapping dose levels 25 μg/kg and 50 μg/kg in children and adults, a difference was not observed between the Cmax and Tmax in adults and pediatrics, but the half-life and AUC were significantly shorter and lower in children, indicating higher rhIL-11 clearance in pediatrics than adults. The AUC in children and adults were 56.7 *vs*. 115 ng·h/mL at 25 μg/kg and 117 *vs.* 242 ng·h/mL at 50 μg/kg, respectively, and the half-lives were 4 *vs.* 8 h at 25 μg/kg and 4.4 *vs.* 8.1 h at 50 μg/kg. The maximum tolerated dose (MTD) of rhIL-11 in children and adolescents was found to be 50 μg/kg/day, which is similar to that reported in adults.

#### 2.6.2. Etanercept

In a population pharmacokinetic study, 69 patients with juvenile rheumatoid arthritis(JRA), aged 4–17 years, received twice weekly subcutaneous injections of 0.4mg/kg etanercept [107]. Sex was a covariate for CL/F, and power exponent of body surface area was found to be 1.41 when normalizing BSA by the typical BSA of 1.071 m^2^. Body weight was found to be a significant covariate for V/F with a typical body weight of 30.8 kg. This analysis justified the body weight based dose adjustment for etanercept in JRA patients. Age (<17 years) was identified as one of the most important covariates on CL in the population pharmacokinetic analysis of pooled data obtained from 10 clinical studies [108]. The correlation between age and CL was no longer apparent for patients aged 17 years and older. Body weight was also found to be a significant covariate for both apparent clearance and volume of distribution in rheumatoid arthritis patients [109]. Gender difference was found in apparent clearance in these adults with a mean level of 0.117 L/h in females and 0.138 L/h in males, but the difference was not statistically significant. A similar trend was found in JRA patients with the population mean CL/F of 0.0576 L/h (95%CI: 0.0525–0.0657 L/h) in females and 0.0772 L/h (95% CI: 0.066–0.0870 L/h) in males [107]. The elimination mechanism of etanercept is not well understood, and there was no appropriate explanation for the gender difference reported both in children and adults. In this JRA patients study, a simulation was conducted to find out whether BSA or body weight adjustment would be a better dosing regimen [107]. To calculate the dose for the BSA based regimen, it was assumed that a patient with the weight of the population median (*i.e.,* 30.8 kg) and a patient with the BSA of the population median (*i.e.,* 1.071 m^2^) received the same total dose of etanercept. Therefore, for example, 11.5 mg/m^2^ (= 0.4mg/kg × 30.8 kg/1.071 m^2^) was chosen to be the dose-per-unit BSA for the BSA based dosage in the simulation. In the middle two quartiles, the body surface area and body weight dosing adjustment yielded similar PK profiles. Interestingly, the simulated PK profiles of the BSA based dosing were slightly higher than body weight based dosing, and the opposite was observed in the highest quartile. The study also concluded that the current body weight based dosing in patients weighing equal to or less than 23 kg may have less drug exposure compared to patients weighing more than 23 kg. However, the pharmacokinetic difference of etanercept was not known to lead to clinical difference in JRA patients.

#### 2.6.3. Enfuvirtide

Enfuvirtide is approved for HIV treatment in adults and dosage recommendations exist for children aged six years or older. The safety and efficacy study of 2.0 mg/kg (maximum 90 mg) subcutaneous enfuvirtide twice daily for 48 weeks was conducted in 52 treatment-experienced, HIV-1-infected pediatric patients (ages 3–16 years) [110]. There was no significant difference observed in the enfuvirtide mean ± SD pharmacokinetic parameters in children (n = 12, ages 5–11 years) and adolescents (n = 13, ages 12–16 years): steady-state Cmax 6.43 ± 2.15 *vs*. 5.88 ± 2.81 μg/mL; Ctrough 2.87 ± 1.49 *vs.* 2.98 ± 1.66 μg/mL; AUC_0-12h_ 56.1 ± 19.4 *vs*. 52.7 ± 27.4 h·μg/mL. There was no meaningful difference in the pharmacokinetic values between children and adolescents. In treatment-experienced HIV-1-infected children (3–12 years), 60 mg/m^2^ subcutaneous enfuvirtide twice daily reported mean single dose AUC_0-12h_ of 56.4 h·μg/mL, which is comparable to AUC_0-12h_ of 55.8 h·μg/mL in adults and also similar to AUC_0-12h_ of 48.7 h·μg/mL in adults after s.c. BID 90 mg enfuvirtide [111,112]. The pediatric study showed that the body weight adjusted dosing in children was independent of age, body weight, body surface area, and sexual maturity. In a population pharmacokinetic analysis study by Zhang *et al*., 43 patients (20 adolescents and 23 children) were included, with a mean age of 11 years, and a mean body weight of 35.7 kg [113]. Body weight was a covariate for CL/F but not V/F. The population parameters CL/F, V/F, and Ka for a 33 kg patient were 1.31 L/h, 2.31 L, and 0.105 h^-1^, respectively. Age did not seem to affect the enfuvirtide exposure. This analysis approves the body weight based enfuvirtide dosing in pediatrics. In HIV-1-infected adults, enfuvirtide reported a small volume of distribution (5.48 L), low clearance (1.4 L/h), and high plasma protein binding (92%). Body weight based dosing (2 mg/kg BID) provides similar pharmacokinetic profiles to those observed with 90 mg BID [114]. Pharmacokinetic parameter CL/F (1.31 L/h for a 33 kg patient) from the pediatric study is comparable to the reported value from a previous study in HIV-1-infected pediatric patients (CL = 1.42 L/h and F = 0.90 for a 21.3 kg patient) [20], and also comparable to the adult population analysis with CL/F of 1.82 L/h for a 70 kg male patient and 1.45 L/h for a 70-kg female patient [115]. The mentioned pediatric enfuvirtide study by Soy *et al* involved 26 children (mean age 8.2 years and range 4.0–12.1 years) [20]. Patient weight was found to have an effect on CL and V, but the effect was not statistically significant. However, their predicted “adult” PK parameters were not comparable with those observed in adults by Zhang *et al* [113]. Additionally, in the plot of CL (L/h) *vs.* weight, even though the data covers a large weight range, it does not seem to capture all differences between children and adults. However, if plotting the Soy *et al* data as CL/kg (L/h/kg) *vs.* weight, the trends seem to be decreasing, but it is unknown whether the trend is statistically significant. 

#### 2.6.4. L-Asparaginase

In a study of pediatrics with acute lymphoblastic leukemia, 271 patients were given 500, 750, 1000, or 2500 IU/m^2^ PEG- L-Asparaginase [116]. After adjusting dose by body surface area, neither the patients’ age (1–17 years) nor the body surface area had any influence on the distribution of Asparaginase activity. The study concluded that normalization of dose based on body surface area was appropriate in the pediatric patients studied. A statistical analysis using linear regression was performed to compare chemotherapy dose modifications in obese and non-obese pediatric patients with acute lymphoblastic leukemia (ALL) [117]. Obese ALL children were reported to have a 7% decrease in the mean relative modification of L-asparaginase compared with non-obese children. The result was statistically significant even after taking into consideration gender, age, race, and study center. It was found that the difference of dose modifications was greater among older children (aged 10–18 years) than small children (aged 2–9 years). It is pointed out that the obesity-driven dose modification among older children is possibly due to higher BSAs and the chemotherapy doses.

## 3. Conclusions

Most of the studies in the current review showed that body weight or BSA dose adjustment produced comparable exposure for proteins and peptides. However, not all pharmacokinetic studies result in promoting dosing adjustment. For basiliximab, a fixed dose of two × 10 mg doses for patients less than 35 kg and a fixed dose of two × 20 mg for patients more than 35 kg was recommended for pediatrics [33]. Children should not be treated as “small adults”. Changes in clearance of proteins and peptides in pediatric patients cannot always be explained by changes in body size. Simply adjusting dose linearly according to the body weight/BSA cannot always achieve desirable exposure in pediatrics. 

Anderson and Holford have proposed that growth and development can be evaluated using readily observable demographic information such as weight and age [118,119,120]. Weight was suggested to be an essential covariate for determining dose in pediatrics. The range of body weight in children is much greater than that in adults and can be 200-fold (0.5–100 kg). An established framework was believed to support the allometry used in pediatric pharmacokinetics. The coefficient exponent of body weight/typical body weight was suggested to be 0.75 for clearance and 1 for volume. Fat free mass may be better than total body weight when variations in fat affect body composition. A sigmoid Emax model was used to describe gradual maturation of clearance from small children to adults. Future issues were suggested in pediatric pharmacokinetics and pharmacodynamics [114]: 1) Determination of *in vivo* maturation of clearance enzyme pathways; 2) Analysis of the placenta concentration to total clearance; 3) Investigation of elimination pathway triggered by birth; 4) Understanding the impact of hormonal changes on clearance pathway in adolescents; 5) Refining PBPK models for children; 6) Further understanding of pharmacodynamic differences between children and adults. 

Overall, the differences of the pharmacokinetics of proteins and peptides in pediatric patients are due to catabolic enzymes, changes in body composition, elimination organs, and receptor mediated endocytosis. The differences lead to changes in the volume of distribution, clearance and absorption of proteins and peptides. The above factors can be dramatically affected by body weight, BSA, height, age, and these covariates may be highly correlated and not mutually exclusive. Due to the complexity of the contributors involved, the direction and extent of the differences are not always readily predictable. Clearance and volume of distribution of proteins and peptides can be higher but also lower when the comparisons are done in children and adults or younger children and older children. In the current review, most of the proteins and peptides show a more rapid body size adjusted clearance (e.g., L/h/kg) in children than in adults, such as alemtuzumab, epoetin, factor VII, factor VIII, and factor XI, while both absolute CL (L/h) and body surface area adjusted CL (L/h/m^2^) of gemtuzumab are smaller in infants than in adults. Enfuvirtide does not provide consistent conclusions from different studies. One pediatric study showed that the pharmacokinetics of body weight adjusted enfuvirtide in children was independent of age, body weight, body surface area, and sexual maturity [110], but from the figure of CL (L/h) *vs.* weight reported by Soy *et al*, the decreasing CL/body weight does not seem to support the body weight adjusted dose of enfuvirtide. Some of the studies showed that a body size adjusted dose for certain proteins and peptides produces comparable exposure in children and adults, and the pharmacokinetics of these products are not affected by age, for example, infliximab, cetuximab, drotrecogin alfa, L-Asparaginase. 

Though there is no obvious similarities for drugs that should not follow simple body size linear adjustment, quit a few monoclonal antibodies are among them. This may due to the fact that monoclonal antibodies are often reported to have nonlinear pharmacokinetics. Basiliximab has less PK variability if 10 mg (weight < 35 kg) and 20 mg (weight > 35 kg) is used in pediatrics. Daclizumab tends to underdose younger patient and overdose larger children. Alemtuzumab and natalizumab underdose children, resulting in non desired clinical outcomes. 

Eating disorders such as anorexia and bulimia are rising among adolescent girls in the United States. On the other side, the rate of obesity in adolescents is also increasing. Anorexia related hospitalizations in children younger than 12 surged 119 percent between 1999 and 2006 [121]. As the pharmacokinetic parameters may be even more complex, simple body weight adjusted dose might not be suitable for such a particular population.

The finding from this review suggest the need to continue the study of proteins and peptides in this particular population, and mechanism based population pharmacokinetic and pharmacodynamic models with consideration of body size and maturity might be helpful in explaining and extrapolating the pharmacokinetics and pharmacodynamics of the studies. Dose adjustment in pediatrics should lead to not only consistent exposure compared with adults, but also decreased intersubject variability in the exposure; only then does it make sense to apply the adjustment. 
